# Au–Ag alloy nanoparticle-incorporated AgBr plasmonic photocatalyst

**DOI:** 10.1038/s41598-020-77062-6

**Published:** 2020-11-17

**Authors:** Shin-ichi Naya, Hiroaki Tada

**Affiliations:** 1 Environmental Research Laboratory, Kindai University, 3-4-1, Kowakae, Higashi-Osaka, Osaka 577-8502 Japan; 2 Department of Applied Chemistry, Faculty of Science and Engineering, Kindai University, 3-4-1, Kowakae, Higashi-Osaka, Osaka 577-8502 Japan

**Keywords:** Chemistry, Nanoscience and technology

## Abstract

A solid-phase photochemical method produces Au–Ag alloy nanoparticles (NPs) with a sharp size distribution and varying composition in AgBr crystals (Au–Ag@AgBr). These features render Au–Ag@AgBr promising as a material for the plasmonic photocatalyst further to provide a possibility of elucidating the action mechanism due to the optical tunability. This study shows that the visible-light activity of Au–Ag@AgBr for degradation of model water pollutant is very sensitive to the alloy composition with a maximum at the mole percent of Au to all Ag in AgBr (y) = 0.012 mol%. Clear positive correlation is observed between the photocatalytic activity and the quality factor defined as the ratio of the peak energy to the full width at half maximum of the localized surface plasmon resonance band. This finding indicates that Au–Ag@AgBr works as a local electromagnetic field enhancement-type plasmonic photocatalyst in which the Au–Ag NPs mainly promotes the charge separation. This conclusion was further supported by the kinetic analysis of the light intensity-dependence of external quantum yield.

The development of technologies contributing to the solution of the energy and environmental issues and improvement in the sanitation are highly desired for the realization of sustainable and safe society. A key material is unambiguously photocatalysts among which plasmonic photocatalysts consisting of plasmonic metal nanoparticle (NP) and semiconductor have recently attracted much attention as a new type of visible-light photocatalysts^1–4^. The plasmonic metal NPs strongly absorb visible light due to the localized surface plasmon resonance (LSPR) originating from the collective oscillation of free electrons coupled with the electromagnetic field of the incident light. The plasmonic photocatalysts can be categorized into the hot-electron transfer (HET)-type and the local electromagnetic field-enhanced (LEFE)-type (or the plasmon-resonant energy-transfer-type)^5^. The former is driven by the LSPR-induced hot electron injection from plasmonic metal NP to semiconductor, and in the latter, the excitation of semiconductor is enhanced by the LSPR-induced intense electromagnetic field. So far, the HET-type plasmonic photocatalysts including Au(core)–Ag(shell) NP-loaded TiO_2_^6^ have mainly been studied. On the other hand, silver-silver halides (Ag–AgX, X = Cl, Br, I) constitute a family of the plasmonic photocatalysts applicable to solar-to-fuel conversions including CO_2_ reduction^7–10^, H_2_ evolution reaction,^11,12^ and N_2_ fixation^13^ in addition to environmental remediation^14^ and sterilization of bacteria^15^. In spite of the high versatility, the action mechanism of the Ag–AgX plasmonic photocatalysts is still elusive, and its accurate understanding would underpin further improvement in the performances. The key to experimentally elucidating the action mechanism of the Ag–AgX plasmonic photocatalysts is the control of the size and composition of Ag NPs affecting the optical properties. Also, the development of the method for measuring the size of Ag NPs in the AgX matrix is necessary. Although transmission electron microscopy (TEM) is usually used for the purpose, the technique cannot be applied to Ag–AgX since it is sensitive to electron-beam irradiation.

Here we report a technique for determining the size of the plasmonic metal NPs in AgBr crystals to show that Au–Ag alloy NPs with a sharp distribution and varying composition can be formed in AgBr crystals (Au–Ag@AgBr) by a solid-phase photochemical method^16^. By taking advantage of the optical tunability of the Au–Ag NPs, we have revealed that the Au–Ag@AgBr plasmonic photocatalyst works as a LEFE-type plasmonic photocatalyst^5^, and the Au–Ag NPs mainly contribute to the charge separation enhancement by the LSPR-induced intense near-field.

## Results and discussion

### Photocatalyst characterization

**Scheme 1:**
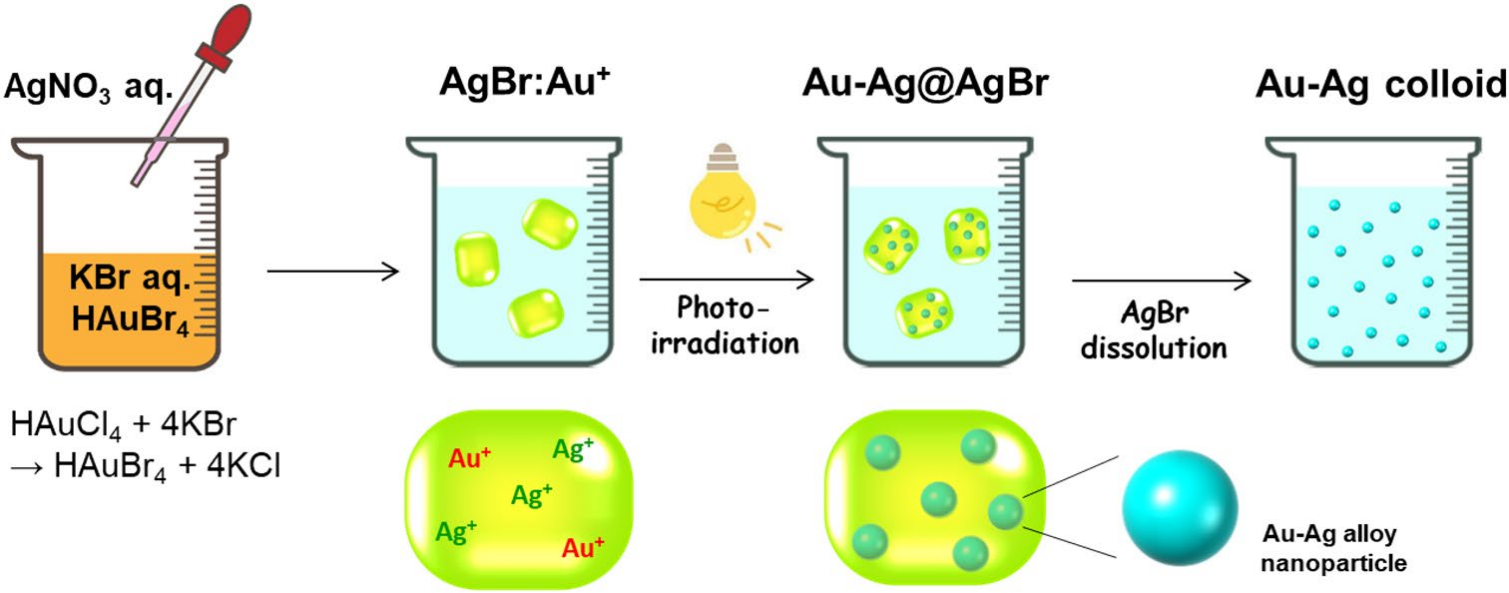
Photocatalyst preparation by the solid-phase photochemical method.

**Figure 1:**
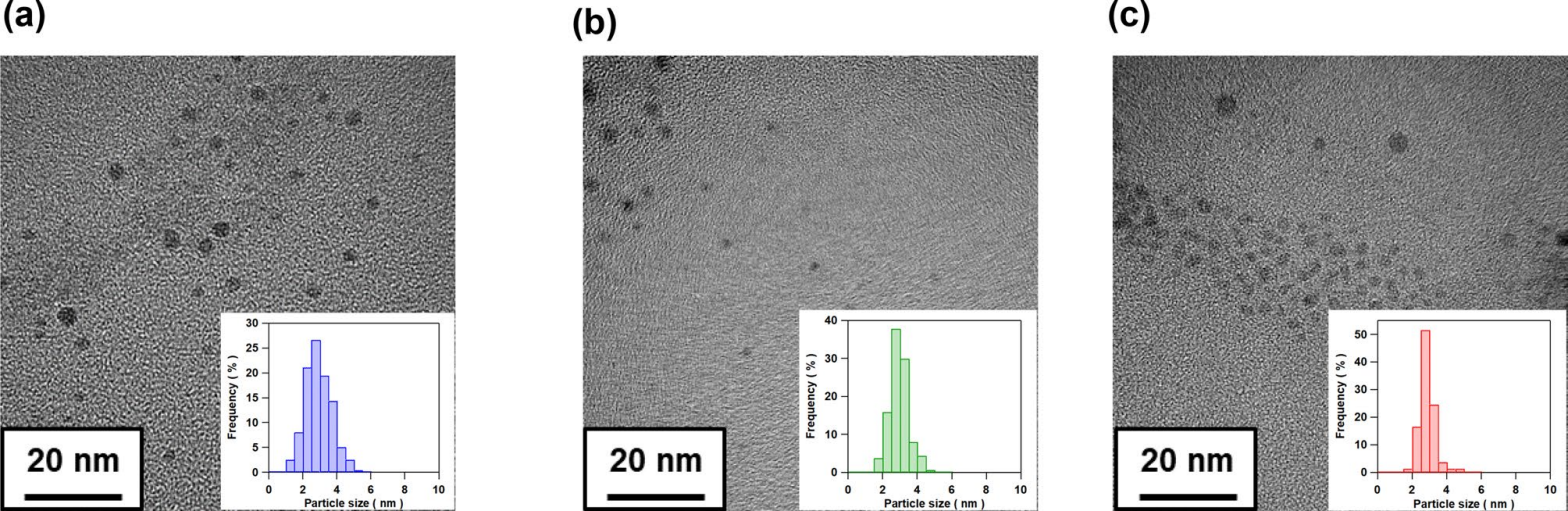
TEM images of the metal NPs obtained by dissolving the AgBr matrix for Au–Ag@AgBr with (**a**) *y* = 0, (**b**) *y* = 0.012 mol%, and (**c**) *y* = 0.159 mol%. Each inset shows the size distribution of metal particles.

Au–Ag alloy nanoparticles (NPs)-incorporated AgBr crystals (Au–Ag@AgBr) with varying composition were synthesized by the solid-phase photochemical method recently developed (Scheme 1)^16^. The amount of Au and Ag in Au–Ag@AgBr were determined by inductively coupled plasma (ICP) spectroscopy. The dose of Au was expressed by *y* (mol%) = (Au mole/all Ag mole in Au–Ag@AgBr) × 100, and controlled at 0 ≤ *y* ≤ 0.159 mol% by the HAuCl_4_ concentration. In the X-ray diffraction (XRD) patterns of the samples with varying *y*, no diffraction peak of Au and/or Ag metal is observed because of the small amount, while the formation of AgBr is clearly confirmed (Supplementaly Fig. S1). The AgBr matrix of Au–Ag@AgBr was selectively dissolved by NaCl aqueous solution with octadecyltrimethylammonium chloride, and the metal particles were observed by TEM. High resolution (HR)-TEM image and energy dispersive X-ray spectroscopy (EDS) line analysis indicate that Au–Ag alloy NP with high crystallinity is generated in the AgBr matrix (Supplementaly Fig. S2). The TEM images of the metal NPs obtained from Au–Ag@AgBr with different Au-dose levels show that uniform nearly spherical metal NPs are produced (Fig. 1, Supplementaly Fig. S3). The mean sizes of metal NPs (*d*) were determined to be *d* = 2.90 nm at *y* = 0, *d* = 2.95 nm at *y* = 0.012 mol%, and *d* = 2.84 nm at *y* = 0.159 mol%, indicating that the *d* is almost independent of *y*. The standard deviation (*σ*) is smaller than 0.75 nm, while the values for the alloy nanoparticles prepared by usual liquid-phase methods are in the range from 1.2 to 2.1 nm^17,18^. The uniformity of Au–Ag metal NPs in the present solid-phase photochemical method probably results from the restriction of the metal particle growth via coalescing particles in the AgBr solid matrix^19^. The sharp size distribution of Au–Ag NPs enables the experimental scrutiny of the action mechanism of the Au–Ag@AgBr plasmonic photocatalyst (vide infra).

### Photocatalytic activity

**Figure 2:**
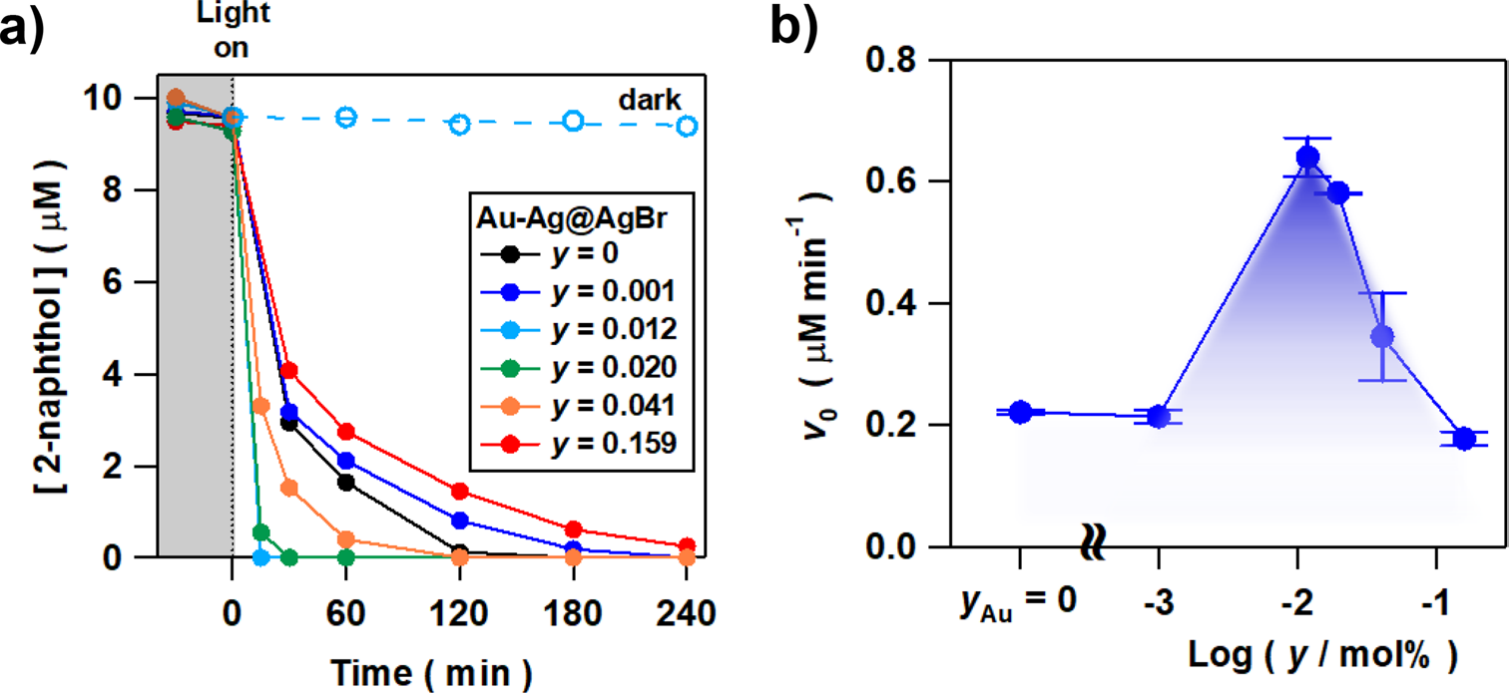
(**a**) Time courses for the degradation of 2-naphthol under visible-light irradiation (*λ* > 400 nm, light intensity *I*_420-485_ = 4.0 mW cm^−2^) by Au–Ag@AgBr with varying *y* and in the dark by using Au–Ag@AgBr (*y* = 0.012 mol%). (**b**) Plots of the initial reaction rate (*v*_0_) and vs. log (*y*/mol%).

2-Naphthol was selected as a model water pollutant since it is industrially used for the production of azo-dyes, also having no absorption in the visible region. After Au–Ag@AgBr particles (20 mg) were added to 10 µM 2-naphthol aqueous solution (20 mL, pH 6.73) and stirred in the dark for 30 min, time evolution for the degradation was traced under visible-light irradiation (λ > 400 nm) at 298 K (Fig. 2a). The light intensity was adjusted so that the intensity integrated from 420 to 485 nm was 4.0 mW cm^−2^ using an actinometer sensitive to the wavelength range. The degradation of 2-naphthol proceeds with irradiation, while its concentration is almost invariant in the dark. The photocatalytic activity of Au–Ag@AgBr strongly depends on the alloy composition. Strikingly, the 2-naphthol disappears within only 15 min at *y* = 0.012 mol%. Several intermediates containing 2,2′-dihydroxy-1,1′-binaphthyl were generated during the irradiation to be finally oxidized to CO_2_ in 68% yield after 24 h irradiation. In this manner, the Au–Ag@AgBr photocatalyst has a strong oxidation power to completely mineralize 2-naphthol by long-time irradiation. To check the catalyst stability, the photocatalytic degradation of 2-naphthol for 15 min was repeated 5 times using Au–Ag@AgBr (*y* = 0.012 mol%) (Supplemental Material Fig. S4). The high degradation yield is maintained after 5-times repetition of the reaction. The photocatalytic activity is almost maintained within an experimental error. Further, ion chromatography and ICP measurements confirmed that no leaching of Ag^+^ and Br^−^ ions from the catalysts to the reaction solution occurs. The initial rate of reaction (*v*_0_) in each system was calculated from the 2-naphthol concentration at the first sampling time (15 or 30 min). The plot of *v*_0_ vs. log (*y*/mol%) shows a volcano-shaped curve with a maximum at *y* = 0.012 mol% (Fig. 2b). The highest *v*_0_ value is greater than that for non-dose sample (*y* = 0) by a factor of ~ 3, while further increase in *y* rapidly decreases the photocatalytic activity.

### Action mechanism of Au–Ag@AgBr

**Figure 3:**
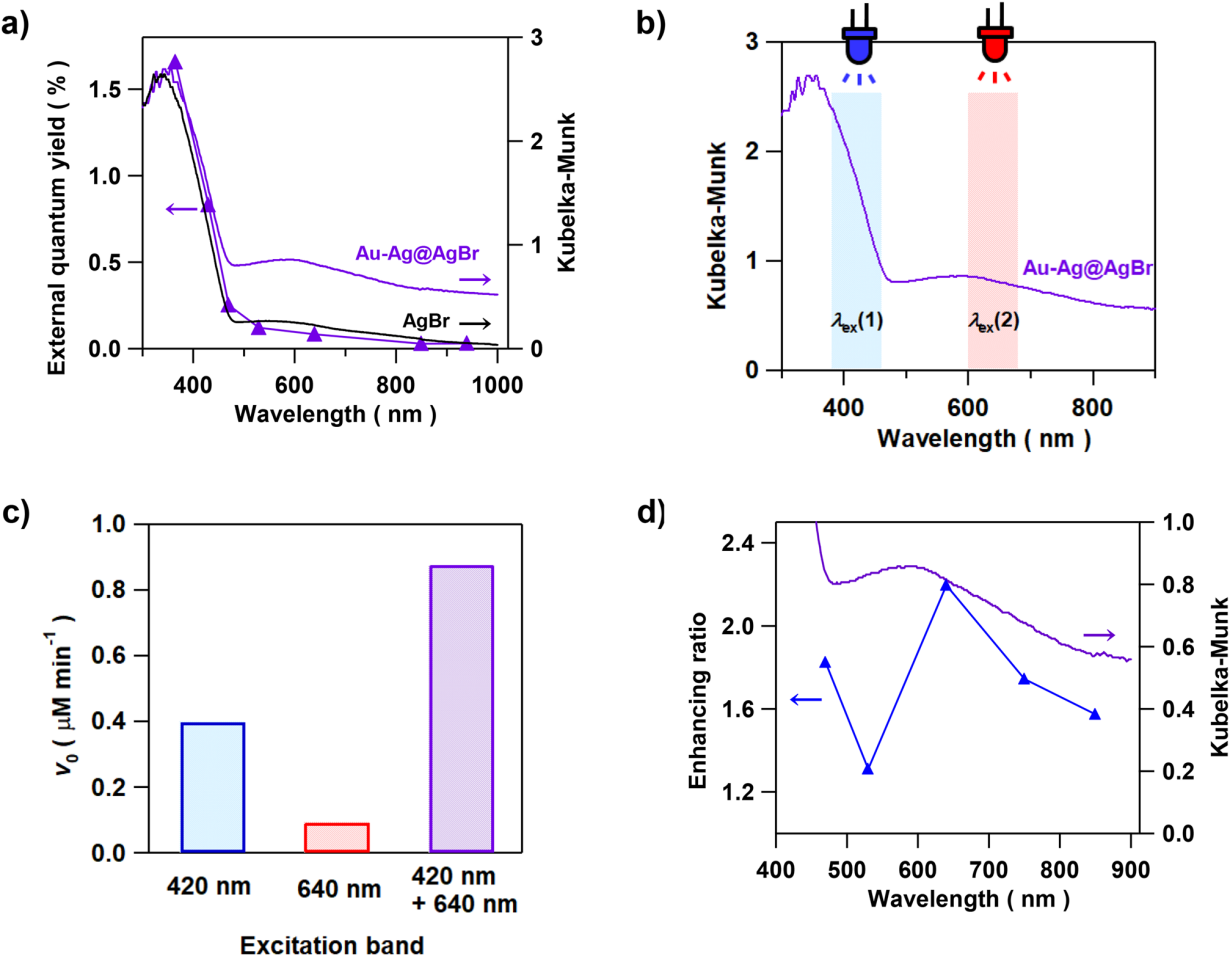
(**a**) Action spectrum of the external quantum yield (*ϕ*_ex_) for the Au–Ag@AgBr (*y* = 0.012 mol%) photocatalyzed degradation of 2-naphthol. For comparison, the absorption spectra of Au–Ag@AgBr (*y* = 0.012 mol%) and AgBr are also shown. (**b**) Excitation band for the 2-naphthol degradation. (**c**) Comparison of the initial reaction rate (*v*_0_) in the Au–Ag@AgBr (*y* = 0.012 mol%) system under different excitation conditions. (**d**) Plots of reaction-enhancing ratio as a function of *λ*_ex_(2) with the *λ*_ex_(1) fixed at 420 ± 40 nm.

AgBr is a semiconductor with the indirect band gap of 2.6 eV^19^. In the absorption spectrum of Au–Ag@AgBr (*y* = 0.012 mol%) (Fig. 3a), the strong absorption band due to the interband transition rises at *λ* ≈ 480 nm, and the broad absorption around 600 nm results from the LSPR of Au–Ag alloy NPs. To gain information about the origin for the Au–Ag alloying effect, the action spectrum analysis was performed for the photocatalytic degradation of 2-naphthol using LEDs with different emission wavelengths. The action spectrum of the apparent quantum yield or external quantum yield (*ϕ*_ex_) bears good resemblance to the absorption spectrum of AgBr with no clear peak corresponding to the LSPR of the Au–Ag NPs (Fig. 3a). This result excludes the hot-electron transfer mechanism from the major route in this reaction because in the case, the action spectrum should have a peak corresponding to the LSPR absorption^20^.

The reaction was further carried out under excitation by light with *λ*_ex_(1) = 420 ± 40 nm or/and *λ*_ex_(2) = 640 ± 40 nm using two LEDs (Fig. 3b). Irradiation of Au–Ag@AgBr at *λ*_ex_(1) and *λ*_ex_(2) almost selectively excites the band gap of AgBr and the LSPR of Au–Ag NPs, respectively. The comparison of the *v*_0_ values for the excitation at *λ*_ex_(1), *λ*_ex_(2), and *λ*_ex_(1) + *λ*_ex_(2) indicates that the excitation at *λ*_ex_(1) causes rapid degradation of 2-naphthol, while the reaction is sluggish under the excitation at *λ*_ex_(2) (Fig. 3c). Simultaneous excitation (*λ*_ex_(1) + *λ*_ex_(2)) greatly enhances the reaction, i.e., the *v*_0_(*λ*_ex_(1) + *λ*_ex_(2)) is much larger than the sum of *v*_0_(*λ*_ex_(1)) and *v*_0_(*λ*_ex_(2)). A similar synergetic effect was previously observed in the Ag NP-deposited AgI system^21^. Further, *λ*_ex_(2) was changed from 470 to 850 nm with the *λ*_ex_(1) fixed at 420 ± 40 nm. Plots of the enhancing ratio defined as *v*_0_(*λ*_ex_(1) + *λ*_ex_(2))/*v*_0_(*λ*_ex_(1)) vs. *λ*_ex_(2) shows that the profile is good resemblance to the LSPR absorption of the Au–Ag NPs (Fig. 3d). Evidently, the Au–Ag@AgBr photocatalyst is driven by the band-gap excitation of AgBr with the activity boosted by the LSPR of incorporated Au–Ag alloy NPs, i.e., Au–Ag@AgBr works as a LEFE-type plasmonic photocatalyst^16^.

The quality factor (*Q*-factor) defined as the ratio of the peak energy (*E*_res_) to the full width at half maximum (*Γ*) of the LSPR band (Eq. 1) expresses the enhancement of the oscillation amplitude of an oscillating system with respect to the driving amplitude^22^.

1 $${\text{Q-factor}} = E_{{{\text{res}}}} /\Gamma$$

**Figure 4:**
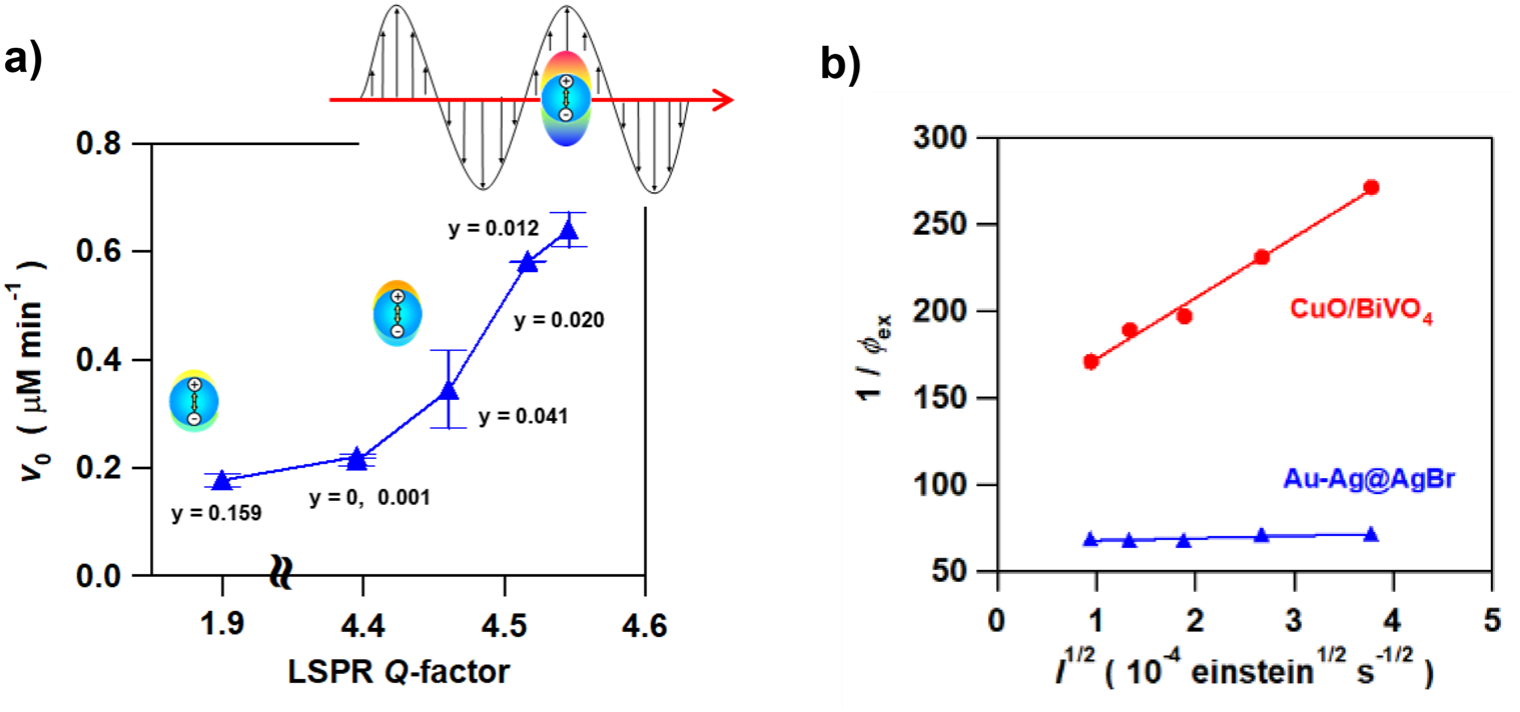
(**a**) Plots of *v*_0_ vs. LSPR *Q*-factor in the Au–Ag@AgBr photocatalyzed degradation of 2-naphthol. (**b**) The plot of ln *v*_0_ vs ln *I* in the same reaction as Fig. 3a where *I* is the intensity of incident light.

For example, the intensity of surface enhanced Raman scattering is proportional to *Q*^4^ ^23–25^, and thus, the Q-factor can be an experimental quantitative indicator for the LEFE-type plasmonic photocatalyst. The absorption spectra of Au–Ag alloy colloids with varying *y* were measured by selectively removing the AgBr matrix, and the *Q*-factor was calculated from Eq. (1) from each *E*_res_ and *Γ*. Interestingly, the plot of *v*_0_ vs. *Q*-factor in the Au–Ag@AgBr systems with varying *y* exhibits good positive correlation between them (Fig. 4a). Thus, the unique *y*-dependence of the photocatalytic activity (Fig. 2b) can be explained in terms of the LEFE effect of the Au–Ag alloy NPs. However, the origin for the increase in *Q*-factor with a slight dose of Au into Ag NP remains unclear since simulations for Au_*x*_–Ag_1−*x*_ alloy NPs at 0 ≤ *x* ≤ 1 by finite-difference time-domain method indicated that the increase in *x* decreases the LEFE factor with the LSPR peak wavelength redshifted^26–30^.

**Scheme 2:**
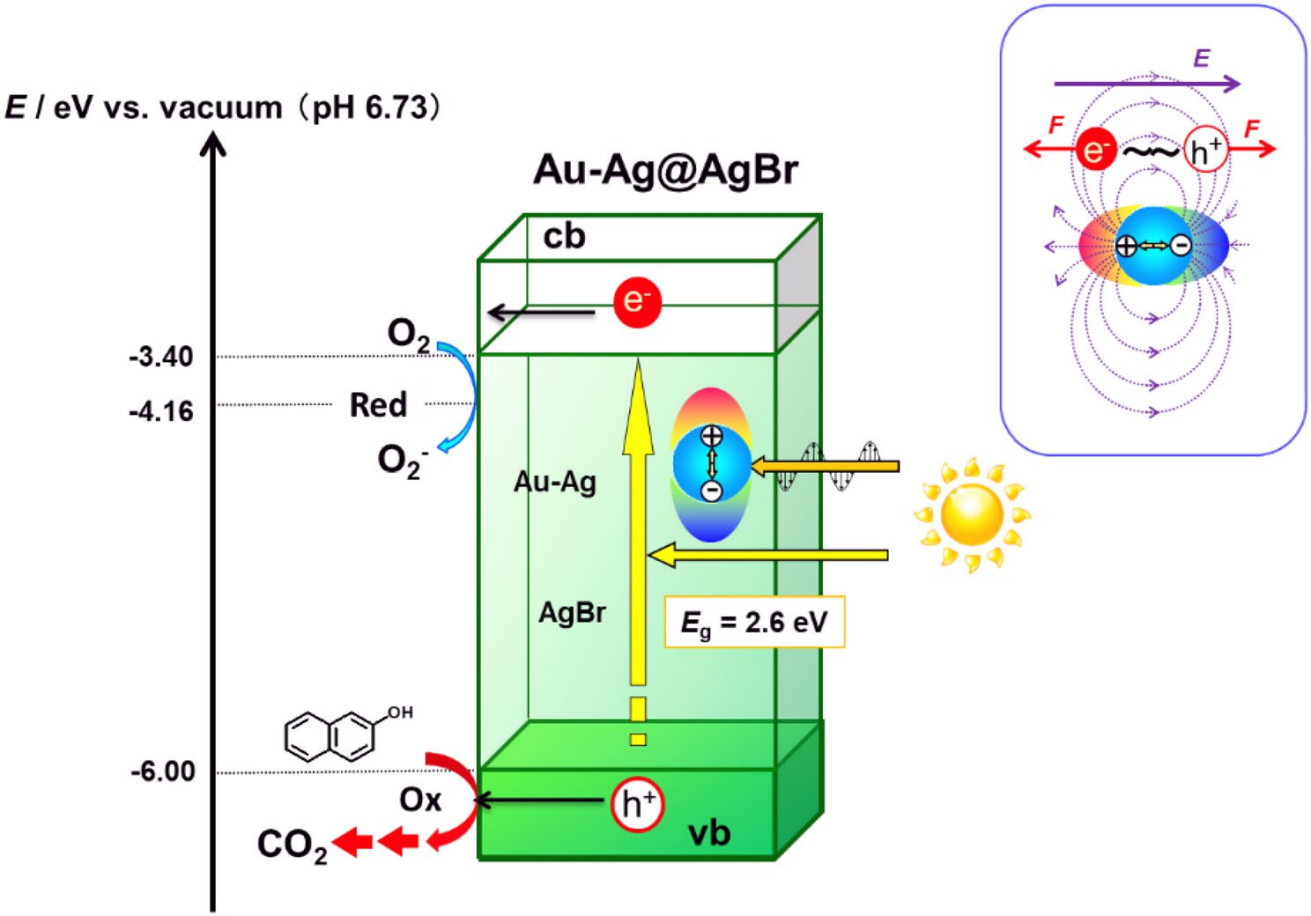
Reaction mechanism proposed for the Au–Ag@AgBr-photocatalyst.

Based on these results, we discuss the action mechanism of Au–Ag@AgBr in the photocatalytic degradation of 2-naphthol (Scheme 2). Visible light (*λ* > 400 nm) excites the electrons in the valence band (vb) of AgBr to the conduction band (cb) simultaneously with the LSPR of Au–Ag alloy NPs. The excited electrons in the cb of AgBr possess a high energy of − 3.40 eV vs. vacuum^19^ to cause oxygen reduction reaction with a redox potential of *E*(O_2_/O_2_^−^) = − 4.16 V vs. vacuum at pH 6.73^31^. The remaining holes in the vb and superoxide radicals can oxidize the adsorbed 2-naphthol finally to CO_2_. The LSPR-induced LEFE effect of Au–Ag alloy NPs can enhance the photoexcitation of AgBr and the charge separation to boost the photocatalytic activity. A previous study on hollow AgI–Ag plasmonic photocatalyst suggests that its high visible-light activity for Rhodamine B degradation results from the increase in the rate of electron–hole pair generation in AgI by the amplified near-field of Ag NP-LSPR^32^. For this scheme to effectively work, significant overlap between the interband transition band of semiconductor and the LSPR of metal NPs is necessary^33^. However, in the Au–Ag@AgBr system, the spectral overlap between them is small, and thus, the charge separation enhancement effect would mainly contribute to the increase in the photocatalytic activity.

In the photocatalytic degradation of 2-naphthol, the application of the steady-state approximation to the excited electrons and holes yields Eq. (2) relating *ϕ*_ex_ and incident light intensity *I* under the conditions of *ϕ*_ex_ <  < *α* (light absorption by photocatalyst) (see Supplemental Material Method). In this case, *αI* expresses the generating rate of electron–hole pairs.

2 $${1}/\phi_{{{\text{ex}}}} \approx \, (k_{{{\text{rec}}}} /k_{{{\text{red}}}} k_{{{\text{ox}}}} \left[ {{2} - {\text{naphthol}}_{{{\text{ad}}}} } \right]\left[ {{\text{O}}_{{{\text{2ad}}}} } \right]\alpha )^{{{1}/{2}}} I^{{{1}/{2}}}$$

where *k*_rec_ is the rate constant of recombination, *k*_red_ is the rate constant of oxygen reduction reaction, and *k*_ox_ is the rate constant for 2-naphthol oxidation.

Linear relation can be expected between 1/*ϕ*_ex_ and *I*^1/2^, and the smaller slope (or smaller ratio of *k*_rec_/*k*_red_*k*_ox_[2-naphthol_ad_][O_2ad_]*α*) indicates the greater charge separation efficiency in the system. We have recently reported that CuO cluster-surface modified BiVO_4_ (CuO/BiVO_4_) semiconductor photocatalyst^34^ is also active for the degradation of 2-naphthol. As predicted, the plots of *ϕ*_ex_ vs. *I*^1/2^ for the Au–Ag@AgBr, and CuO/BiVO_4_ systems provide straight lines with the slopes of 1.4 × 10^4^ and 3.5 × 10^5^ einstein^−1/2^ s^1/2^, respectively (Fig. 4b). The much smaller slope in the Au–Ag@AgBr system further supports the conclusion that the Au–Ag NPs in AgBr greatly enhance the charge separation.

## Conclusions

The solid-phase photochemical method yields Au–Ag alloy NPs with a sharp size distribution and varying composition in AgBr crystals (Au–Ag@AgBr). A slight amount of Au-dosing gives rise to a drastic increase in the photocatalytic activity for the degradation of 2-naphthol used as a model water pollutant. Clear positive correlation between the photocatalytic activity and the LSPR-quality factor of Au–Ag NPs indicates that the striking Au–Ag alloy effect is mainly induced by the charge separation enhancement due to the intense local electric field generated at the interface between Au–Ag NP and AgBr. These conclusions would also be valid for other silver halide-based systems, underpinning their further development as the plasmonic photocatalysts for the solar-to-fuel conversion and environmental purification.

## Methods

### Catalyst preparation and characterization

The catalysts were prepared by the solid-phase photochemical method recently developed^14^. KBr (12 mmol) and HAuCl_4_ was dissolved into distilled water (80 mL). To the solution, AgNO_3_ aqueous solution (0.1 M, 40 mL) was dropwise slowly by means of perista pump with 0.5 mL min^−1^. After stirring at room temperature for 1 h, the generating particles were collected by centrifugation. The sample was washed with distilled water several times, and dried in vacuo to form AgBr:Au^+^. The particles (0.1 g) were added to MeOH (20 mL), and re-dispersed by ultrasonication. UV-light (*λ* = 365 nm, the light intensity integrated from 310 to 420 nm (*I*_310–420_) = 4.0 mW cm^−2^) was irradiated by using LED at room temperature for 15 min. The resulting particles were washed with distilled water several times, and dried in vacuo to yield Au–Ag@AgBr. The Au amount was determined by inductively coupled plasma spectroscopy (ICPS-7000, Shimadzu). The *y* value was varied in the range at 0 ≤ *y* ≤ 0.159 mol% by changing the concentration of HAuCl_4_. Transmission electron microscopy (TEM) observation was carried out by means of JEOL JEM-2100F with an applied voltage of 200 kV. By using UV-2600 spectrometer (Shimadzu) with integrating sphere unit (Shimadzu, ISR-2600Plus), diffuse reflectance UV–Vis-NIR spectra of the samples were measured at room temperature. The reflectance (*R*_∞_) was obtained with respect to BaSO_4_ as a reference, and the Kubelka–Munk function [*F*(*R*_∞_) =  = (1 − *R*_∞_)^2^/2R_∞_] was used for the calculation of the relative absorption coefficient.

### Photocatalytic decomposition of 2-naphthol

Au–Ag@AgBr (20 mg) was added to an aqueous solution of 2-naphthol (10 µM, 20 mL) with 1% acetonitrile. Acetonitrile with a wide potential window was added for complete dissolution of 2-naphthol. The suspension was stirred in the dark for 30 min, and then, illumination was carried out by using a 300 W Xe lamp (HX-500, Wacom) with a cut off filter L-42 (*λ* > 400 nm, AGC TECHNO GLASS) in a double jacket type reaction cell. The cell was kept at 25 °C by circulating thermostated water through an outer jacket around the cell. The light intensity (*I*_420–485_) was adjusted to 4.0 mW cm^−2^ using an actinometer sensitive to the wavelength range from 420 to 485 nm. The 2-naphthol concentration was determined by high-performance liquid chromatography (Prominence, Shimadzu) [measurement conditions: *λ* = 223 nm; Shim-pack CLC-ODS (*ϕ* 4.6 mm × 150 mm) (Shimadzu); mobile phase H_2_O : MeOH = 3 : 7; flow rate = 1 mL min^−1^;]. The amount of CO_2_ generated was measured by gas chromatography (GC-2014, C-R8A with methanizer MTN-1 (Shimadzu)) [measurement conditions: N_2_ flow rate = 50 mL min^−1^; column = Porapak-Q 80–100 (GL science)].

## Supplementary information

Supplementary Information.
