# Supplementary material for: Au–Ag alloy nanoparticle-incorporated AgBr plasmonic photocatalyst
Source: Sci Rep. 2020 Nov 17;10:19972. doi: 10.1038/s41598-020-77062-6 (PMC7673129; doi:10.1038/s41598-020-77062-6)

**Au-Ag Alloy Nanoparticle-Incorporated AgBr Plasmonic Photocatalyst**

Shin-ichi Naya,^a^ Hiroaki Tada ^b^*

*^a^ Environmental Research Laboratory, Kindai University, 3-4-1, Kowakae, Higashi-Osaka, Osaka 577-8502, Japan.*

*^b^ Department of Applied Chemistry, Faculty of Science and Engineering, Kindai University, 3-4-1, Kowakae, Higashi-Osaka, Osaka 577-8502, Japan*

AUTHOR INFORMATION

* To whom correspondence should be addressed: TEL: +81-6-6721-2332, FAX: +81-6-6727-2024,

E-mail: [h-tada@apch.kindai.ac.jp](mailto:h-tada@apch.kindai.ac.jp).

**Methods**

**Photocatalytic reaction efficiency**

To obtain the action spectrum, 2-naphthol decomposition was carried out under similar conditions under illumination of monoclomatic light using LED. The apparent quantum yield or external quantum efficiency (*φ*_ex_) was calculated by Eq. S1 by assuming a one-electron oxidation.

**_ex_ (%) = {(molecule number of 2-naphthol degraded)/(incident photon number)} × 100

= {(molecule number of 2-naphthol degraded)/( *I* × *A* × *λ* × 3600 /*hc* )} × 100 (S1)

where *I* is incident light intensity (W cm^-2^), *A* is incident area (10 cm^2^), *λ* is light wavelength, *h* is Planck constant, *c* is the speed of light.

**Light intensity dependency**

CuO-cluster surface modified BiVO_4_ (CuO/BiVO_4_, Cu loading amount = 1.96 ions nm^-2^) were prepared by the reported procedures.^S1^ Au-Ag@AgBr (*y* = 0.012 mol%) or CuO/BiVO_4_ (5 mg) was added to an aqueous solution of 2-naphthol (10 μM, 5 mL) with 1% acetonitrile. Acetonitrile was added for the complete dissolution of 2-naphthol. After stirring in the dark for 15 min, illumination was carried out by using a 300 W Xe lamp (HX-500, Wacom) with a cut off filter L-42 (*λ* > 400 nm, AGC TECHNO GLASS) and neutral density filter in a double jacket type reaction cell. The cell was kept at 25^o^C by circulating thermostated water through an outer jacket around the cell. The light intensity was changed from 0.25 to 4.0 mW cm^-2^. The 2-naphthol concentration was determined by high-performance liquid chromatography (Prominence, Shimadzu) [measurement conditions: *λ* = 223 nm; Shim-pack CLC-ODS (*φ* 4.6 mm × 150 mm) (Shimadzu); mobile phase H_2_O : MeOH = 3 : 7; flow rate = 1 mL min-1;]. The amount of CO_2_ generated was measured by gas chromatography (GC-2014, C-R8A with methanizer MTN-1 (Shimadzu)) [measurement conditions: N2 flow rate = 50 mL min-1; column = Porapak-Q 80-100 (GL science)].

S1. Kunimoto, T., Naya, S. & Tada, H. Copper oxide cluster surface modification-induced multiple electron oxygen reduction reaction on bismuth vanadate under visible-light Irradiation. *J. Electrochem. Soc.* **167**, 116523/1-7 (2020).

**Appendix for Eq. 2**

The processes for the photocatalytic degradation of 2-naphthol can be divided into the following elemental steps in which S denotes semiconductor-based photocatalyst. Irradiation of S with energy larger than the band gap and light intensity of *I* promotes the electrons in the valence band (vb) to the conduction band (cb) at the rate of *Iα *(Eq. S2).

S + *h* → e_cb_^-^ + h_vb_^+^ (S2)

Most of the electron-hole pairs are lost by the recombination with the rate of *k*_rec_ (Eq. S3).

e_cb_^-^ + h_vb_^+^ → S (S3)

The excited cb-electrons reduce O_2_ adsorbed on S with the rate constant of *k*_red_ (Eq. S4).

O_2ad_ + e_cb_^-^ → O_2ad_ ^-^ (S4)

The vb-holes oxidize 2-naphthol adsorbed on S with the rate constant of *k*_ox_ (Eq. S5). In this case, the oxidation of 2-naphthol by superoxide anions was neglected.

2-naphthol+ h_vb_^+^ → oxidation product (S5)

Application of the steady-state approximation to the cb-electrons and vb-holes gives Eq. S6 and S7.

*Iα* = *k*_ox_ [h_vb_^+^][2-naphthol_ad_] + *k*_rec_ [e_cb_^-^][ h_vb_^+^] (S6)

*k*_red_[e_cb_^-^][O_2ad_] = *k*_ox_ [h_vb_^+^][2-naphthol_ad_] (S7)

The quantum yield (*φ*_ex_) can be defined by the ratio of the rate of oxidation (or reduction) to the incident light intensity *I* (Eq. S8).

**_ex_* * = (d[2-naphthol_ad_]/d*t*)/*I* (S8)

Combination of Eqs. S6, S7, and S8 yields Eq. 2 under the conditions of *α* >> *φ*_ex_.

1/*φ*_ex_= (*k*_rec_/*k*_red_*k*_ox_[2-naphthol_ad_][O_2ad_]*α*)^1/2^*I*^1/2^ (2)

**Figure S1.** XRD patterns of Au-Ag@AgBr with varying *y*.


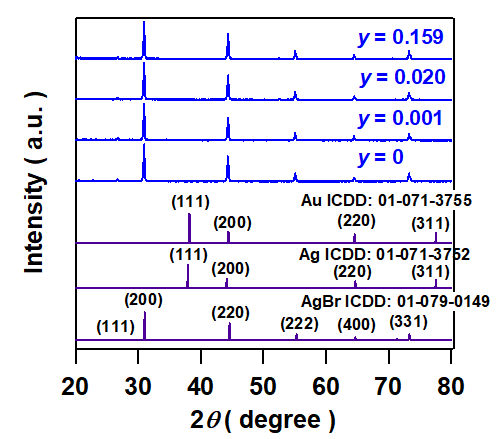


**Figure S2.** (a) HR-TEM image (a) and EDS line analysis (b) of a metal NP obtained by dissolving the AgBr matrix for Au-Ag@AgBr with *y* = 0.041 mol%

**(a)**

**(b)**


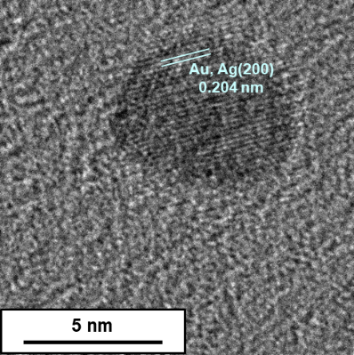

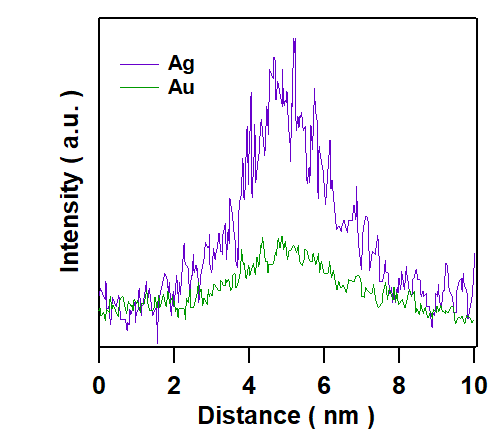


**Figure S3.** TEM images of the metal NPs obtained by dissolving the AgBr matrix for Au-Ag@AgBr with (a) *y* = 0.001, (b) *y* = 0.020 mol%, and (c) *y* = 0.041 mol%). Each inset shows the size distribution of metal particles.

**(a)**

**(b)**

**(c)**


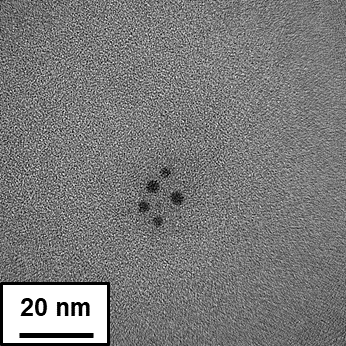

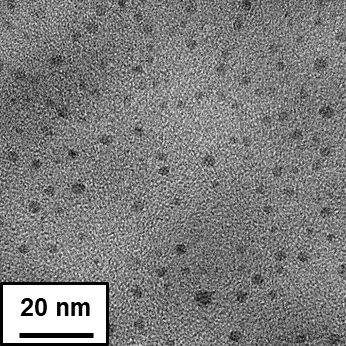

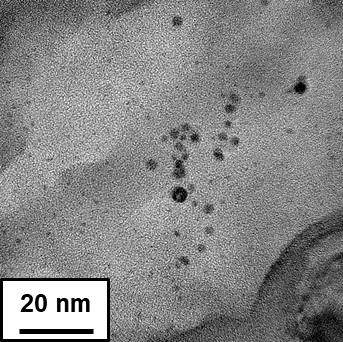

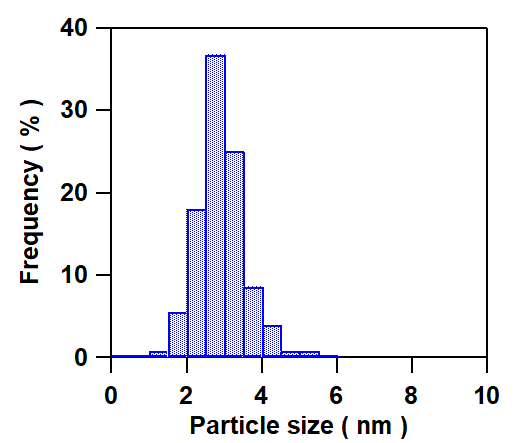

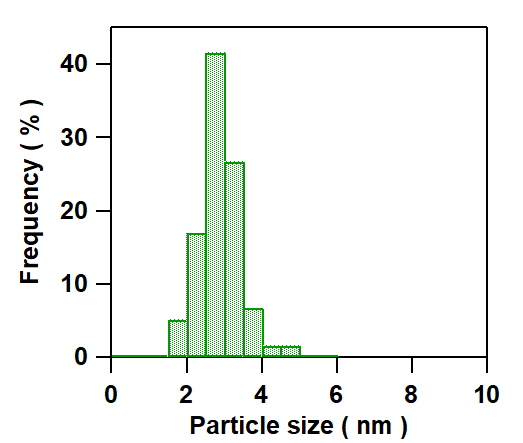

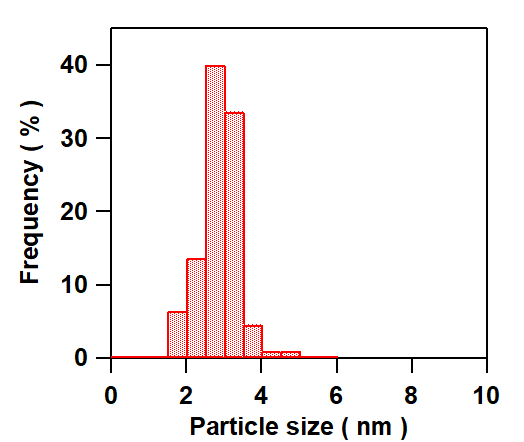


Figure S4. Repeated photocatalytic degradation of 2-naphthol by Au-Ag@AgBr (*y* = 0.012 mol%) with illumination for 15 min.


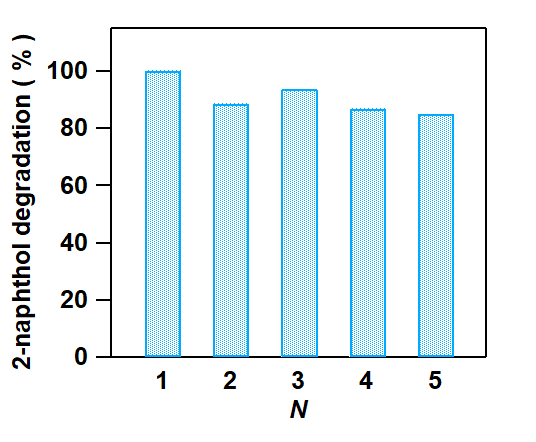

Supplement: Supplementary file 1 — Supplementary Information. [file 41598_2020_77062_MOESM1_ESM.docx]
